# HMTase Inhibitors as a Potential Epigenetic-Based Therapeutic Approach for Friedreich’s Ataxia

**DOI:** 10.3389/fgene.2020.00584

**Published:** 2020-06-05

**Authors:** Mursal Sherzai, Adamo Valle, Nicholas Perry, Ester Kalef-Ezra, Sahar Al-Mahdawi, Mark Pook, Sara Anjomani Virmouni

**Affiliations:** ^1^Ataxia Research Group, Division of Biosciences, Department of Life Sciences, College of Health and Life Sciences, Brunel University London, Uxbridge, United Kingdom; ^2^Energy Metabolism and Nutrition, Research Institute of Health Sciences (IUNICS) and Health Research Institute of Balearic Islands (IdISBa), University of Balearic Islands, Palma de Mallorca, Spain; ^3^Biomedical Research Networking Center for Physiopathology of Obesity and Nutrition (CIBERobn), Instituto de Salud Carlos III, Madrid, Spain; ^4^Division of Cancer Biology, The Institute of Cancer Research, London, United Kingdom

**Keywords:** FRDA, Friedreich ataxia, frataxin, FXN, GAA repeat, HMTase inhibitor

## Abstract

Friedreich’s ataxia (FRDA) is a progressive neurodegenerative disorder caused by a homozygous GAA repeat expansion mutation in intron 1 of the frataxin gene (*FXN*), which instigates reduced transcription. As a consequence, reduced levels of frataxin protein lead to mitochondrial iron accumulation, oxidative stress, and ultimately cell death; particularly in dorsal root ganglia (DRG) sensory neurons and the dentate nucleus of the cerebellum. In addition to neurological disability, FRDA is associated with cardiomyopathy, diabetes mellitus, and skeletal deformities. Currently there is no effective treatment for FRDA and patients die prematurely. Recent findings suggest that abnormal GAA expansion plays a role in histone modification, subjecting the *FXN* gene to heterochromatin silencing. Therefore, as an epigenetic-based therapy, we investigated the efficacy and tolerability of two histone methyltransferase (HMTase) inhibitor compounds, BIX0194 (G9a-inhibitor) and GSK126 (EZH2-inhibitor), to specifically target and reduce H3K9me2/3 and H3K27me3 levels, respectively, in FRDA fibroblasts. We show that a combination treatment of BIX0194 and GSK126, significantly increased *FXN* gene expression levels and reduced the repressive histone marks. However, no increase in frataxin protein levels was observed. Nevertheless, our results are still promising and may encourage to investigate HMTase inhibitors with other synergistic epigenetic-based therapies for further preliminary studies.

## Introduction

Friedreich ataxia (FRDA) is the most common autosomal recessive ataxia. It is caused by homozygous GAA repeat expansion mutation within intron 1 of the frataxin (*FXN*) gene (Campuzano et al., 1996), which induces *FXN* gene silencing and hence reduced expression of the essential mitochondrial protein frataxin (Campuzano et al., 1997). Frataxin insufficiency leads to iron-sulfur cluster protein deficits, increased oxidative stress, mitochondrial iron accumulation, and ultimately cell death, with primary sites of pathology being the large sensory neurons of the dorsal root ganglia (DRG) and the dentate nucleus of the cerebellum (Koeppen, 2011). Clinically, the outcome is progressive spinocerebellar neurodegeneration, hypertrophic cardiomyopathy and a high prevalence of diabetes due to pancreatic β-cell dysfunction (Cnop et al., 2012), with premature death occurring in early adulthood (Pandolfo, 2009). Although there is currently no effective treatment for FRDA, advances in research of its pathogenesis have led to a wide range of therapeutic strategies that are being tested in clinical trials. These include the use of antioxidants such as idebenone, EPI-A0001 and pioglitazone; iron chelators such as deferiprone; and frataxin-increasing compounds such as erythropoietin (EPO) and histone deacetylase (HDAC) inhibitors (Schulz et al., 2009). Of these, the synthetic coenzyme Q10 analog, idebenone, has undergone the most intensive testing of any compound, including two large Phase III clinical trials, placebo-controlled NICOSIA (National Institutes of Health Collaboration with Santhera in Ataxia) study of 48 children (Lynch et al., 2010) and IONIA (Idebenone Effects on Neurological ICARS Assessments) study of 70 pediatric participants (Lagedrost et al., 2011) over 6 months. While a positive effect was observed in the secondary outcome of left ventricular heart mass in one study (Mariotti et al., 2003), these trials have failed to show alleviation of the neurological symptoms associated with FRDA (Kearney et al., 2012). Therefore, there is still a high unmet clinical need to develop effective FRDA therapies.

Novel epigenetic-based frataxin-increasing therapies are considered to be among the most promising approaches. Although DNA methylation is considered to be a stable epigenetic modification, post-translational modifications of histone proteins play more flexible roles in transcriptional regulation. Acetylation and methylation of histones at lysine (and arginine) residues are highly dynamic and are involved in several neurological disorders (Ziemka-Nalecz et al., 2018; Wang and Liu, 2019). Histone acetylation at lysine residues is regulated by two distinct families of enzymes with opposing action, histone acetyltransferases (HATs) and histone deacetylases (HDACs). Similarly, histone lysine methylation is controlled by histone methyltransferases (HMTases) and histone demethylases (e.g., LSD1 and JmjC), which have been linked to a number of cellular processes including DNA repair, replication, transcriptional activation, and repression (Kouzarides, 2007). Transcriptional repression of genes is associated with hypoacetylation of certain histone residues, primarily H3K9, together with increased methylation of histone residues, such as H3K9me3, H3K27me3, and H4K20me3 (Kourmouli et al., 2004; Martin and Zhang, 2005; Jenuwein, 2006).

In FRDA cells, such histone modifications have been identified within the *FXN* gene, predominantly at the region immediately upstream of the expanded GAA repeats, indicating that the *FXN* gene is subject to heterochromatin silencing (reviewed in Sandi et al., 2013). These findings have encouraged the investigation of epigenetic-based therapies, in particular the use of histone deacetylase (HDAC) inhibitors, to reverse *FXN* gene silencing (reviewed in Gottesfeld et al., 2013; Sandi et al., 2013). In fact, several compounds, such as the 2-aminobenzamide HDAC inhibitor 109 and nicotinamide, have shown interesting outcomes reversing the *FXN* gene silencing in cell and mouse models of FRDA (Sandi et al., 2011; Chutake et al., 2016). Subsequently, RG2833 – the formulated form of 109 – has been taken forward to a phase I clinical trial showing increased levels of *FXN* mRNA and H3K9 acetylation in peripheral blood mononuclear cells from FRDA patients (Soragni et al., 2014). Although targeting histone acetylation marks appears promising, no studies have yet explored the therapeutic potential of targeting histone methylation. The class III HDAC inhibitor, nicotinamide – which is able to increase *FXN* expression in FRDA cell and mouse models – has also been shown to decrease the levels of H3K9me3 and H3K27me3 at the *FXN* locus (Chan et al., 2013).

As an additional epigenetic-based therapeutic approach for FRDA therapy, we chose to investigate the use of HMTase inhibitors to improve *FXN* expression in FRDA. HMTase enzymes are lysine- or arginine-specific and usually modify only one particular histone residue. On the basis of pharmacological outcome and efficacy in other diseases such as cancer (Kubicek et al., 2007; McCabe et al., 2012), we chose two HMTase inhibitors, BIX0194 (G9a-inhibitor) and GSK126 (EZH2-inhibitor) to specifically target and reduce H3K9me2/3 and H3K27me3 levels, respectively, in FRDA human primary fibroblasts.

## Materials and Methods

### Cell Lines and Culture Conditions

FRDA and healthy control fibroblasts were grown in DMEM medium with 10% FBS and 1% penicillin-streptomycin (all from Invitrogen) in 5% CO_2_ at 37°C. Human and mouse primary cell lines were treated with 1 nM–10 μM of BIX01294 and GSK126 alone or in combination in triplicate for 72 h. Mouse fibroblast cell lines were established from YG8sR FRDA and Y47R control mouse models as previously described (Anjomani Virmouni et al., 2015).

### PrestoBlue^®^ Cell Viability Assay

Cells were cultured in a 24 well culture plate for 24 h and washed once with PBS followed by adding fresh medium containing 1 nM–10 μM of BIX01294 and/or GSK126 (in DMSO, final concentration 0.1% v/v) in triplicates for 72 h. Control cells were also cultured and treated simultaneously with the equivalent volume of DMSO at 0.1% finalconcentration (v/v). Cells were incubated for 72 h and PrestoBlue^®^ reagent (Invitrogen) was added to a 1x final concentration followed by incubating the cells for further 3 h. Upon entering a living cell, PrestoBlue^®^ reagent is reduced from resazurin, a blue compound with no intrinsic fluorescent value, to resorufin which is red in color and highly fluorescent. Conversion is proportional to the number of metabolically active cells and therefore can be measured quantitatively. The fluorescence intensity was then measured using xMark^TM^ Microplate Absorbance Spectrophotometer (Bio-Rad) with an excitation wavelength of 570 nm and emission wavelength of 600 nm.

### Quantitative RT-PCR

Total RNA was isolated from cells using the Trizol (Invitrogen) method and cDNA was then prepared by using AMV reverse transcriptase (Invitrogen) with oligo(dT)_20_ primers following the manufacturer’s instructions. Levels of *FXN* and *HPRT* mRNA expression were assessed by quantitative RT–PCR using a QuantStudio 7 Flex Real-Time PCR instrument and SYBR^®^ Green (Applied Biosystems) with the following primers: *FXN*-h-forward 5′-CAGAGGAAACGCTGGACTCT-3′, *FXN*-h-reverse 5′ AGCCAGATTTGCTTGTTTGGC-3′, *HPRT*-h-forward 5′-GGTGAAAAGGACCCCACGA-3′, *HPRT* -h-reverse 5′-TCAAGGGCATATCCTACAACA-3′, *Fxn*-m-forward 5′-TTGAAGACCTTGCAGACAAG-3′, *Fxn*-m-reverse 5′-AGCCAGATTTGCTTGTTTGG-3′, *Hprt*-m-forward 5′- ATGAAGGAGATGGGAGGCCA-3′, *Hprt*-m-reverse 5′- TCCA GCAGGTCAGCAAAGAA -3′. Assays were performed in triplicate in at least two independent experiments. Human Endogenous Control Gene Panel was used to assess the off-target effects of the HMTase inhibitors on global gene expression by qRT-PCR using specific Primer sets (TATAAbiocenter).

### Immunoblot Analysis

Cells were treated with 100 nM BIX01294, 2 μM GSK126 or combination of both for 72 h. Cells were washed with ice-cold PBS and lysed on ice for 30 min with cell lysis buffer (Cell Signaling Technology, 9803) supplemented with 400 μM PMSF protease inhibitor (Cell Signaling Technology, 8553). Lysates were subjected to centrifugation at 12,000 g for 30 min at 4°C and protein concentrations were determined using the BCA protein assay reagent (Thermo Scientific). 50 μg of protein lysates were boiled for 10 min and subjected to SDS-PAGE electrophoresis using 4–15% precast gels (Bio-Rad, 567-1084). Densitometry was calculated using the Image Lab Software 5.2.1 (Bio-Rad). Antibodies used in this study are as follows: anti-frataxin (1:250, ab113691, Abcam), β-actin (1:1000, A2066, Sigma), goat anti-Rabbit HRP (1:5000, P0448, Dako) and goat anti-Mouse HRP (1:5000, P0447, Dako).

### Frataxin Dipstick Assay

Protein concentration was quantified by BCA assay and levels of frataxin protein were measured by lateral flow immunoassay with the Frataxin Protein Quantity Dipstick Assay Kit (MitoSciences, Eugene, OR, United States) according to the manufacturer’s instructions (Willis et al., 2008). Signal intensity was measured with a Hamamatsu ICA-1000 Immunochromatographic Reader (MitoSciences).

### Histone Methyltransferase Activity Assay

Cells were treated with 100 nM BIX01294, 2 μM GSK126 or combination of both for 72 h, followed by nuclear extract preparations using the EpiQuik^TM^ Nuclear Extraction Kit (Epigentek), according to the manufacturer’s instructions. Subsequently, HMTase activities were evaluated using the EpiQuik^TM^ HMTase activity assay kits (for H3K9, P-3003-96 and for H3K27, P-3005-96, Epigentek) according to the manufacturer’s instructions. This assay is based on the principle that the HMTase enzyme G9a and EZH2, transfers a methyl group from S-Adenosyl methionine (Adomet) to lysine 9 and 27 of histone H3, respectively. The level of methylated histone H3K9/H3K27 is then recognized with a high-affinity antibody, which is directly proportional to enzyme activity. This was quantified through horseradish peroxidase (HRP) conjugated secondary antibody-color development system. The resulting absorbance was measured at 450 nm using the xMark^TM^ Microplate reader (Bio-Rad).

### Chromatin Immunoprecipitation-qPCR Assay

Histone modifications at the 5′UTR promoter of *FXN* gene were detected by ChIP analysis in FRDA and control cells. This procedure was performed by using ChIP qPCR kit (Chromatrap) with an acetylated H3 (Lys9) (06-942, Merck Millipore), trimethyl-H3 (Lys9) (07-442, Merck Millipore), and trimethyl-H3 (Lys27) (07-449, Merck Millipore), antibody on formaldehyde cross-linked samples. DNA was then sheared by sonication, followed by immunoprecipitation. For each experiment, normal rabbit serum (SIGMA) was used as a negative control. After reversal of cross-linking, quantitative RT–PCR amplification of the resultant co-immunoprecipitated DNA was carried out with SYBR^®^ Green in a QuantStudio 7 Flex Real-Time PCR instrument (Applied Biosystems) using the following primers; h-*FXN*-pro-forward 5′-AAGCAGGCTCTCCATTTTTG-3′ and h-*FXN*-pro-reverse 5′- CGAGAGTCCACATGCTGCT-3′. The data were from three independent chromatin preparations, with each experiment done in triplicate.

### Statistical Analyses

For statistical analysis, the unpaired two-tailed Student’s *t*-test were used to assess the significance of the differences between group data with a significance value set at *P* < 0.05.

## Results

### Effects of HMTase Inhibitors on Primary Fibroblasts Viability

**FIGURE 1:**
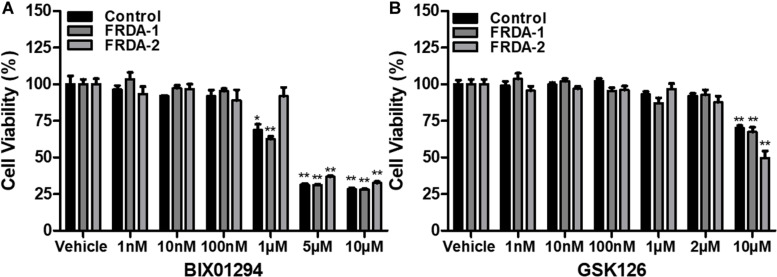
Cell viability analysis following 72 h HMTase inhibitor treatment. **(A)** BIX01294 and **(B)** GSK126 treatment analysis in human FRDA (GM03816 and GM04078) and normal fibroblasts (GM07492). The mean value of all data was normalized to the PrestoBlue reduction of vehicle treated cells (set at 100%). Error bars indicate SEM and values represent mean ± SEM (*n* = 3). Asterisks indicate statistically significant differences between drug and vehicle treated cell lines, assessed by unpaired two-tailed Student’s *t*-test (*P < 0.05, **P < 0.01).

The safety and cellular tolerability of BIX01294 and GSK126 treatment were determined by cell viability assay. Human and mouse primary cell lines were treated with BIX01294 (1 nM–10 μM) and GSK126 (1 nM–10 μM) in triplicate for 72 h. Fibroblast cells tolerated BIX01294 treatment with concentrations ranging from 1 to 100 nM, whereas, higher concentrations significantly decreased their viability (Figure 1A and Supplementary Figure 1A). In comparison to BIX01294 treatment, cells were generally less sensitive to GSK126 treatment (Figure 1B and Supplementary Figure 1B). Both normal and FRDA human fibroblasts indicated no significant change in viability with 1 nM to 2 μM GSK126 treatment (Figure 1B), although cell viability was significantly reduced following treatment with 10 μM GSK126. A similar pattern was also observed in mouse fibroblasts treated with GSK126, however, FRDA cells showed reduced tolerance with 2 μM GSK126 (*P* < 0.05) (Supplementary Figure 1B). These results gave a valuable indication of the optimal compound dosing *in vitro* required for subsequent molecular analysis to determine the efficacy of BIX01294 and GSK125 for FRDA therapy.

### The Effect of HMTase Inhibitors on Frataxin Expression

**FIGURE 2:**
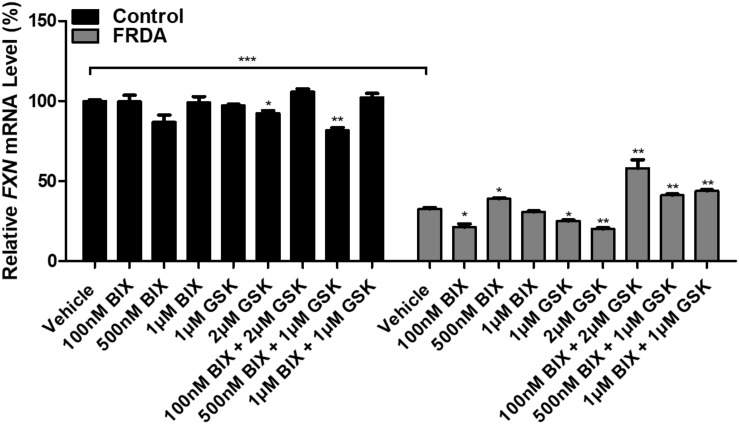
qRT-PCR analysis indicating the relative *FXN* mRNA levels following treatment with BIX01294 and GSK126 alone or in combination in human primary fibroblasts (FRDA, GM03816 and normal, GM07492). Each result displayed is the mean of two independent experiments and the *FXN* mRNA levels of each sample were normalized to *HPRT* gene as an endogenous control. The values were expressed as a ratio to the vehicle treated samples of normal fibroblasts. Error bars indicate SEM and values represent mean ± SEM (*n* = 3). Asterisks indicate significant differences between drug and vehicle treated cell lines, assessed by unpaired two-tailed Student’s *t*-test (*P < 0.05, **P < 0.01, ***P < 0.001).

In order to test the effects of BIX01294 and GSK126 on *FXN* gene reactivation, normal and FRDA human primary fibroblasts were treated with 100 nM, 500 nM, and 1 μM of BIX01294 and 1 and 2 μM of GSK126. The cells were treated with the drug(s) either individually or in combination over a period of 72 h. *FXN* mRNA levels in control cells were generally unaffected by treatments, whether alone or in combination. In FRDA cells, treatment with BIX01294 did not significantly increase *FXN* gene expression levels, except for 500 nM concentrations, which induced a modest increase of 19% (*P* < 0.05) (Figure 2). Similarly, individual treatments of GSK126 did not have a substantial effect on *FXN* mRNA expression levels in FRDA cells, where in fact a significant decrease is seen with 1 μM (22%, *P* < 0.05) and 2 μM (38%, *P* < 0.01) concentrations. However, all combination treatments of BIX01294 and GSK126 in FRDA cell lines were shown to significantly increase *FXN* mRNA levels. The highest significant increase of 88% (*P* < 0.01) was observed with combination treatment of 100 nM BIX01294 and 2 μM GSK126, followed by 34% (*P* < 0.01) increase with 1 μM BIX01294 and 1 μM GSK126 treatment, then 26% (*P* < 0.01) increase with 500 nM BIX01294 and 1 μM GSK126 treatment. This suggests that simultaneous inhibition of both H3K9me2/3 and H3K27me3 with BIX01294 and GSK126, respectively, is beneficial in reversing the histone modifications and in activating the *FXN* gene. These results were confirmed in an additional FRDA cell line, however, interestingly this effect was more predominant in this cell line showing higher level of *FXN* expression compared to the control when treated with the combination of both inhibitors (Supplementary Figure 2). Similar to human fibroblasts, the mouse primary fibroblasts were treated either individually or in combination with 1 and 100 nM of BIX01294 and 100 nM and 1 μM of GSK126 for 72 h. Generally, no significant changes in *FXN* gene expression levels were observed in FRDA mouse cells after individual treatments with BIX01294 and GSK126 (Supplementary Figure 1C). However, similar to human FRDA fibroblasts, combination treatments of 100 nM BIX01294 with 100 nM GSK126 and 100 nM BIX01294 with 1 μM GSK126, both have significantly increased the *FXN* mRNA expression levels in FRDA mouse cells by 16% (*P* < 0.01) and 37% (*P* < 0.01), respectively (Supplementary Figure 1C).

**FIGURE 3:**
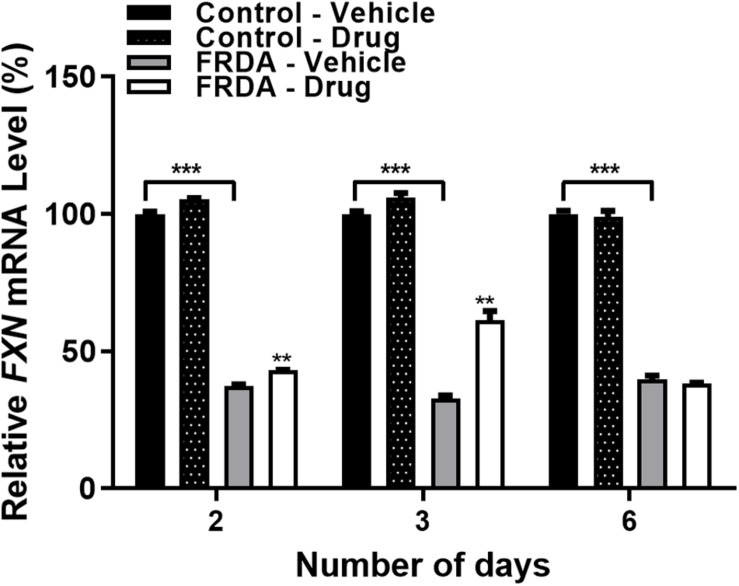
qRT-PCR analysis indicating the relative *FXN* mRNA levels following combination treatment with BIX01294 (100 nM) and GSK126 (2 μM) in human primary fibroblasts (FRDA, GM03816 and normal, GM07492) for different time points. For this treatment, the cell culture medium was replaced with fresh medium supplemented with the drug every 3 days. Mean *FXN* mRNA levels of each sample were normalized to *HPRT* gene as an endogenous control. Values were expressed as a ratio to the vehicle treated samples of normal fibroblasts at the corresponding time point. Error bars indicate SEM and values represent mean ± SEM (*n* = 3). Asterisks indicate significant differences between drug and vehicle treated cell lines, assessed by unpaired two-tailed Student’s *t*-test (**P < 0.01, ***P < 0.001).

After determining the synergistic effect with 100 nM BIX01294 and 2 μM GSK126 as the optimum drug dosing, we then investigated the effect of this combination treatment on *FXN* gene expression levels with different time points (2, 3, and 6-day) in FRDA cell lines (Figure 3 and Supplementary Figure 1D). We noted a gradual increase in *FXN* transcription after 2 and 3-day treatments by 15% (*P* < 0.01) and 88% (*P* < 0.01) in human FRDA cell lines, respectively. Consistent with our results in the human cell lines, 3-day treatment indicated an increase in the *FXN* gene expression by 37% (*P* < 0.001) in FRDA mouse cell lines (Supplementary Figure 1D).

**FIGURE 4:**
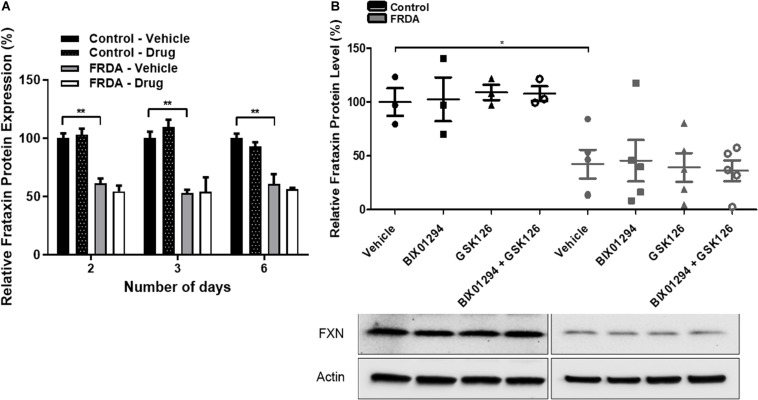
Frataxin expression levels. **(A)** Dipstick immunoassay of frataxin protein in primary fibroblasts (FRDA, GM03816 and normal, GM07492) following synergistic treatment with BIX01294 (100 nM) and GSK126 (2 μM) for different time points. Values are expressed as percentage of the vehicle treated samples of normal fibroblasts at the corresponding time point. **(B)** Western blot analysis indicating the relative frataxin protein expression levels in human fibroblasts (FRDA, GM04078 and FA3, and normal GM08399 and GM08333) following synergistic treatment with BIX01294 (100 nM) and GSK126 (2 μM) for 3 days. Values were normalized to actin levels and were expressed as percentage of the vehicle treated samples of normal fibroblasts. Representative Immunoblots are shown. Error bars indicate SEM and values represent mean ± SEM (*n* = 3). Asterisks indicate significant differences between drug and vehicle treated cell lines, assessed by unpaired two-tailed Student’s *t*-test (*P < 0.05, **P < 0.01).

To determine the change in frataxin protein expression, combination treatments were carried out with 100 nM BIX01294 and 2 μM GSK126 for different time points (2–6 days). The levels of frataxin protein were determined by dipstick assay. As expected, FRDA cells show significantly reduced frataxin protein levels compared to healthy counterparts, but no significant change was observed after drug treatment at any time point (Figure 4A and Supplementary Figure 1E). Western blot analysis further confirmed our dipstick assay results (Figure 4B), suggesting that other post-translational mechanisms may be playing a role in regulation of frataxin protein levels.

### Inhibition of the HMTase G9a and EZH2 by BIX01294 and GSK126

**FIGURE 5:**
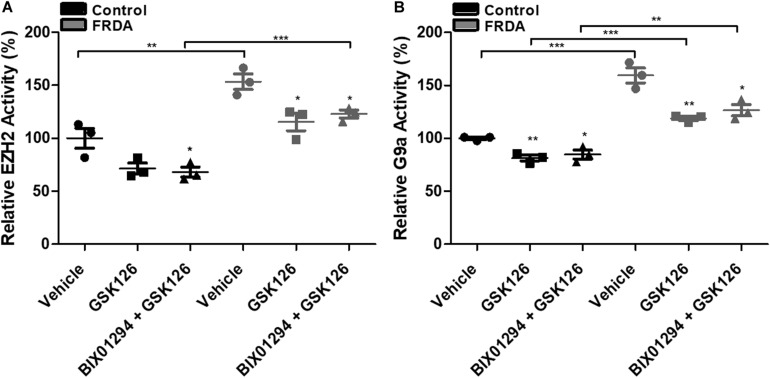
HMTase enzymatic activity analysis in human fibroblasts (FRDA, GM03816 and normal, GM07492) treated individually and in combination with BIX01294 (100 nM) and GSK126 (2 μM). **(A)** EZH2 and **(B)** G9a activity levels. The values were expressed as a ratio to the vehicle treated samples of normal fibroblasts. Error bars indicate SEM and values represent mean ± SEM (*n* = 3). Asterisks indicate significant differences between drug and vehicle treated cell lines, assessed by unpaired two-tailed Student’s *t*-test (*P < 0.05, **P < 0.01, ***P < 0.001).

To assess the inhibitory effects of BIX01294 and GSK126, we investigated the enzymatic activity levels of EZH2 and G9a, respectively, following treatment in human primary fibroblasts. We treated the cells with 100 nM BIX01294 and 2 μM GSK126 either individually or in combination for 72 h. Consistently, the EZH2 activity was significantly higher by 53% (*P* < 0.01) in vehicle treated FRDA fibroblasts as compared to normal fibroblasts (Figure 5A). However, 2 μM GSK and a combination treatment of 2 μM GSK126 + 100 nM BIX01294 in FRDA fibroblasts have shown to significantly reduce the EZH2 activity by 28% (*P* < 0.05) and 20% (*P* < 0.05), respectively. Similarly, FRDA fibroblasts were shown to have a 59% significantly higher G9a enzymatic activity as compared to normal fibroblasts (Figure 5B). This activity was significantly reduced by 25% (*P* < 0.01) and 20% (*P* < 0.01) with 100 nM BIX01294 and combination treatment of 2 μM GSK126 + 100 nM BIX01294, respectively. There was no significant change in EZH2 and G9a activity levels between individual and synergistic drug treatment in FRDA cells. This suggest that the combination treatment of BIX01294 and GSK126 does not interfere with or potentiate the drugs’ specific inhibitory effects. Furthermore, a similar change in EZH2 and G9a enzymatic activity was also observed in normal fibroblasts after treatment, suggesting that BIX01294 and GSK126 might have a non-disease specific inhibitory effect.

### Treatment With HMTase Inhibitors Decreases Repressive H3K9/H3K27 Marks at FXN Promoter

**FIGURE 6:**
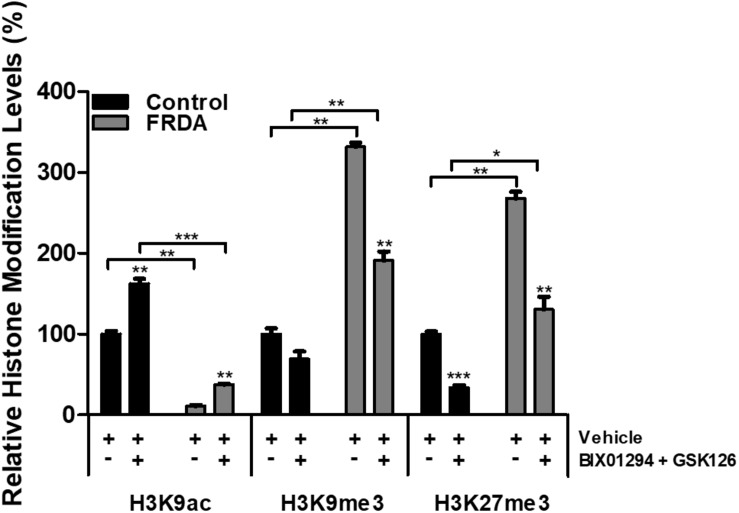
Histone modification changes in the *FXN* 5′UTR promoter region, after synergistic treatment with BIX01294 (100 nM) and GSK126 (2 μM) for 72 h in human FRDA (GM03816) and normal (GM07492) fibroblasts. The values were expressed as a ratio to the vehicle treated samples of normal fibroblasts. Error bars indicate SEM and values represent mean ± SEM (*n* = 3). Asterisks indicate significant differences between drug and vehicle treated cell lines, assessed by unpaired two-tailed Student’s *t*-test (*P < 0.05, **P < 0.01, ***P < 0.001).

To examine the effects of BIX01294 and GSK126 on histone modifications, human primary fibroblasts were treated with a combination of 100 nM BIX01294 and 2 μM GSK126 for 72 h, followed by performing ChIP assay, to determine the histone modification changes in the *FXN* 5′UTR promoter region. Immunoprecipitation with anti-H3K9ac antibody revealed a significant reduction (*P* < 0.01) in FRDA fibroblasts treated with vehicle as compared to normal fibroblasts (Figure 6). Interestingly, the combination treatment with BIX01294 and GSK126 increased H3K9ac in both FRDA and control cell lines (*P* < 0.001). Along with histone hypoacetylation, both lysine 9 and lysine 27 of histone H3 were highly methylated in FRDA cell lines (H3K9me3 and H3K27me3, *P* < 0.01). However, after drug treatment, the methylation levels significantly decreased in H3K9me3 (*P* < 0.01) and H3K27me3 (*P* < 0.01). Nonetheless, an increase in H3K9ac and decrease in H3K9me3 and H3K27me3 levels is also observed in normal fibroblasts after drug treatment, which correlates well with the change in EZH2 and G9a enzymatic activity (Figure 6). Overall, the change in histone modification after drug treatment in FRDA fibroblasts did not reach the regular levels seen in normal fibroblasts.

### Off-Target Effects of HMTase Inhibitors on Gene Expression

**FIGURE 7:**
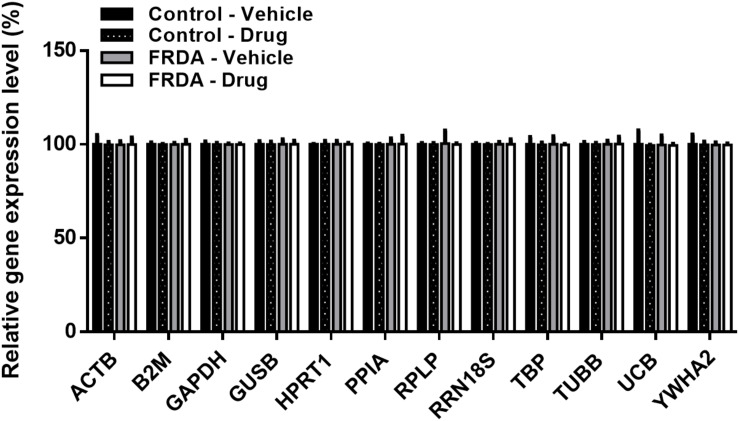
Relative change in the expression of a panel of human endogenous control genes in primary fibroblasts (FRDA, GM03816 and normal, GM07492), following BIX01294 (100 nM) and GSK126 (2 μM) combination treatment. Values were expressed as a ratio to the vehicle treated samples of normal fibroblasts. Error bars indicate SEM and values represent mean ± SEM (*n* = 3). ACTB, beta-actin; B2M, beta-2-microglobulin; GAPDH, glyceraldehyde-3-phosphate dehydrogenase; GUSB, beta-glucuronidase; HPRT1, hypoxanthine-guanine phosphoribosyltransferase; PPIA, cyclophillin A; RPLP, 60S acidic ribosomal protein P0; RRN18S, 18S rRNA; TBP, TATAA-box binding protein, TUBB, beta-tubulin, UCB, ubiquitin C; YWHA2, tyrosine 3/tryptophan 5-monooxygenase activation protein zeta.

Like any epigenetic-based therapies, HMTase inhibitors are expected to induce a widespread effect on gene expression by altering global histone modification levels, and thus have a potential off-target effects. Therefore, to evaluate the possibility of BIX01294 and GSK126 off-target effects in FRDA, we treated human fibroblasts with a combination treatment of the two drugs (100 nM BIX01293 and 2 μM GSK126), which had exerted a change in *FXN* gene expression levels (Figures 3, 4), for 72 h. We then quantitatively measured changes in the expression of a panel of endogenous control genes by qRT-PCR in human fibroblasts. The results obtained show that there is no significant change in any of the genes explored in human fibroblasts after treatment (Figure 7). This suggests that BIX01294 and GSK126 exerts minimal off-target effects outside of *FXN* gene regulation.

## Discussion

It has been reported that 98% of FRDA patients have a homozygous GAA trinucleotide repeat expansion within the first intron of the *FXN* gene, leading to reduced expression of frataxin (Campuzano et al., 1996). Although the mechanism by which the GAA repeat expansion leads to decreased levels of frataxin are currently unknown, it is generally accepted that FRDA may be caused by a heterochromatin-mediated silencing effect of the *FXN* gene (Saveliev et al., 2003; Festenstein, 2006). In support of this hypothesis, differential DNA methylation in FRDA patients accompanied by various histone modifications have been identified within the vicinity of the expanded GAA repeats and near the promoter region of the *FXN* gene. This includes elevated methylation of histone residues, such as H3K9me2/3 and H3K27me3, with hypoacetylation of H3K9 (Herman et al., 2006; Greene et al., 2007; Al-Mahdawi et al., 2008; De Biase et al., 2009). Such DNA and histone modifications can be reversed, representing a suitable target for epigenetic-based therapy. Moreover, since the expanded GAA repeat in FRDA does not alter the amino acid sequence of frataxin, gene reactivation would be of therapeutic benefit (Sandi et al., 2014a). In this study, we have demonstrated the *in vitro* feasibility of two HMTase inhibitors, BIX01294 (G9a-inhibitor) and GSK126 (EZH2-inhibitor), to potentially increase frataxin expression, by reducing histone methylation levels at the *FXN* locus. Notably, 72 h treatment with BIX01294 or GSK126 alone did not significantly enhance *FXN* gene expression in FRDA fibroblasts. In fact, a dose-dependent decrease in *FXN* gene expression level is seen with GSK126 treatments. Previously, it was reported that chemical inhibition of G9a with BIX01294 treatment showed to decrease H3K9 methylation at the *FXN* locus but failed to up-regulate *FXN* to a significant high level (Punga and Buhler, 2010). A possible explanation for this could be that H3K9 methylation may have a redundant role or it may cooperate with another heterochromatin mark in silencing the *FXN* gene. It is interesting to note, that on the silenced *FXN* locus there is presence of both H3K9 and H3K27 methylation at high levels. H3K9 methylation is associated with HP1 mediated silencing of highly heterochromatinised satellite repeats, whereas H3K27 is linked to polycomb-mediated silencing of formerly euchromatin genes. Typically, these two marks do not overlap in the mammalian genome (Yandim et al., 2013). Therefore, it can be hypothesized that there is a cooperation between the H3K9 and H3K27 methylation marks on the *FXN* locus. Indeed, a study in 2003 reported an accumulation of H3K27me3 in SUV39H double null cells may partially substitute for lack H3K9me3 histone marks (Peters et al., 2003).

In accordance with this, combination treatment with BIX01294 and GSK126 was shown to promote a safe induction of *FXN* mRNA expression levels in FRDA fibroblasts, predominantly after a 3-day treatment period. This indicates that simultaneous inhibition of G9a and EZH2, which targets H3K9me2/3 and H3K27me3 repressive histone marks, may have beneficial effect, to some extent, in increasing *FXN* gene expression levels in FRDA. However, frataxin dipstick and western blot analyses revealed that frataxin protein expression levels remained unaffected after combined treatment with BIX01294 and GSK126. This suggests that there may be other post-translational mechanisms at play, affecting either the *FXN* mRNA stability or frataxin protein translation, stability or degradation that will require further investigation. Interestingly, we also identified significantly increased levels of EZH2 and G9a activity in FRDA human fibroblasts as compared to normal fibroblasts. This agrees with previous proposals that stalled RNAPII, during RNA:DNA hybrid formation, may be recruiting high levels of HMTases to methylate histones locally and reducing *FXN* gene transcription, as a defense mechanism (Yandim et al., 2013). Furthermore, following individual and combination treatment of BIX01294 and GSK126, the G9a and EZH2 levels were significantly reduced, respectively, in both the normal and FRDA human fibroblasts. This suggests that the drugs target and inhibit their corresponding HMTase activities in a non-disease specific manner. Moreover, significantly increased H3K9me3 and H3K27me3 levels, alongside decreased H3K9ac levels, were seen in the *FXN* 5′UTR promoter region in FRDA fibroblasts, as previously reported (De Biase et al., 2009; Sandi et al., 2014a). However, after combined treatment of BIX01294 and GSK126, a non-cell type specific reduction in H3K9me3 and H3K27me3, and an increase in H3K9ac was seen, which correlated well with the changes in G9a and EZH2 levels.

Since epigenetic therapies are likely to alter gene expression more widely, we selected a panel of endogenous control genes to assess whether BIX01294 and GSK126 exert significant off-target effects. Although our gene panel was limited and, of note, did not include any known cancer-related genes, we did not observe any off-target effects following combination treatment. Of the selection of genes investigated, TBP, USB1, and HPRT1 are known to have lower expression levels. ChIP-sequencing signals from ENCODE/SYDH in UCSE Genome Browser^1^ reveal that generally these genes have higher histone acetylation marks near the promoter region, with elevated H3K36me3 enrichment alongside lower H3K9me3 and H3K27me3 enrichments throughout the gene (Kent et al., 2002). However, no studies have reported that these genes can be affected by HMTase inhibitors.

Overall, our results indicated that a combination treatment of BIX01294 and GSK126 may be effective in increasing the *FXN* gene expression levels in FRDA, by simultaneously targeting H3K9me3 and H3K27me3 repressive marks. However, based on our findings of frataxin protein levels after drug treatment, *in vivo* animal studies are not proposed at this stage. Compared to other epigenetic-based therapies, the use of HMTase inhibitors is still highly underexplored in FRDA. Since larger expanded GAA repeats are highly associated with heterochromatin mediated *FXN* gene silencing, it is crucial to carry out future *in vitro* studies using patient-derived cells with higher GAA repeats, and possibly different cell culture systems (Sandi et al., 2014b). Nevertheless, HMTase inhibitors should still be pursued for further preclinical studies, perhaps with other synergistic epigenetic-based compounds, such as HDAC inhibitors and DNMT inhibitors. Similar to HMTase inhibitors, HDAC inhibitors could potentially reduce epigenetic silencing of an affected gene by targeting the heterochromatin state. In FRDA, the HDAC inhibitors 109/RG2833 and nicotinamide (vitamin B3) have shown the most promising results in restoring frataxin to normal levels by increasing histone acetylation at the *FXN* locus (Nageshwaran and Festenstein, 2015; Burk, 2017). Moreover, alongside abnormal histone modification, numerous studies have reported an increase in DNA methylation levels in the pathogenic *FXN* alleles. Thus far, no studies have reported the effects of DNA demethylation agents in treating FRDA. Recent reports investigating the TNR disorder fragile X syndrome (FXS), have shown promising results using the DNMT inhibitor, 5-aza-CdR, either alone or in combination with HDAC inhibitors (Chiurazzi et al., 1999) or with HMTase inhibitors (Kumari and Usdin, 2016) to effectively reduce the FMR1 promoter hypermethylation and reinstating mRNA and protein levels to normal in FXS patient cells. Therefore, it would be interesting to investigate the synergistic effects of HMTase inhibitors with HDAC inhibitor compounds and/or DNMT inhibitors in the reactivation of *FXN* gene transcription *in vitro* and subsequently in FRDA mouse models (Anjomani Virmouni et al., 2014, 2015; Sandi et al., 2014b).

In conclusion, the evaluation of therapeutic agents for FRDA has rapidly advanced in the last few decades, with the finding of numerous pharmacological agents at different stages of development. Our study encourages the use of simultaneous administration of two or more epigenetic-based drugs for further preliminary studies to improve disease phenotype in FRDA.

## Data Availability Statement

All datasets generated for this study are included in the article/Supplementary Material.

## Author Contributions

SA-M, AV, MP, and SA conceived and designed the study. MS, SA-M, NP, AV, MP, and SA wrote the manuscript. All authors performed the experiments and read and approved the manuscript.

## Conflict of Interest

The authors declare that the research was conducted in the absence of any commercial or financial relationships that could be construed as a potential conflict of interest.

## Footnotes

1 genome.ucsc.edu
