# Supplementary material for: HMTase Inhibitors as a Potential Epigenetic-Based Therapeutic Approach for Friedreich’s Ataxia
Source: Front Genet. 2020 Jun 5;11:584. doi: 10.3389/fgene.2020.00584 (PMC7291394; doi:10.3389/fgene.2020.00584)
Supplement: Supplementary file 1 [file Data_Sheet_1.docx]

Supplementary Material
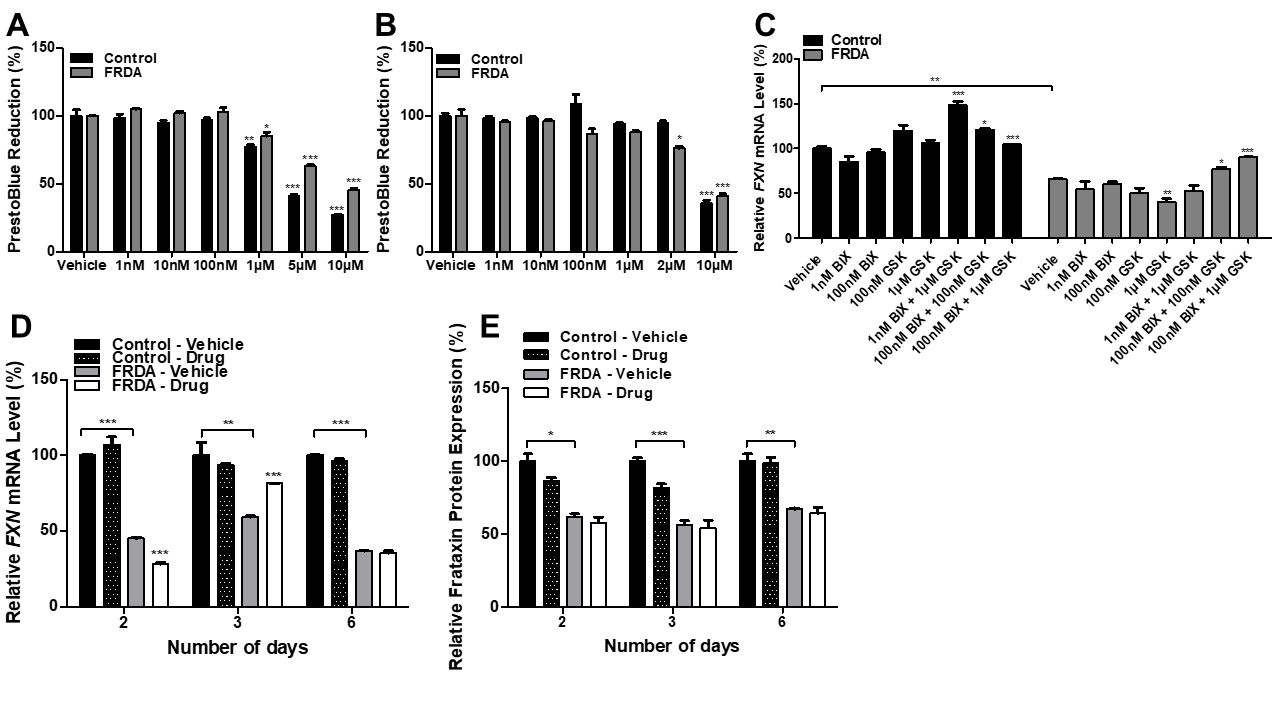


**Supplementary Figure 1** Analysis of HMTase inhibitor treatment in mouse FRDA and normal fibroblasts. Cell viability analysis following 72 hour treatment with **(A)** BIX01294 and **(B)** GSK126 in mouse FRDA (YG8sR) and normal fibroblasts (Y47R). The mean value of all data was normalised to the PrestoBlue reduction of vehicle treated cells (set at 100%). **(C)** qRT-PCR analysis indicating the relative *FXN* mRNA levels following treatment with BIX01294 and GSK126 alone or in combination in mouse primary fibroblasts (FRDA, YG8sR and normal, Y47R). Each result displayed is the mean of two independent experiments and the *FXN* mRNA levels of each sample were normalised to *HPRT* gene as an endogenous control*.* The values were expressed as a ratio to the vehicle treated samples of normal fibroblasts. **(D)** qRT-PCR analysis indicating the relative *FXN* mRNA levels following combination treatment with BIX01294 (100nM) and GSK126 (2µM) in mouse primary fibroblasts (FRDA, YG8sR and normal, Y47R) for different time points. For this treatment, the cell culture medium was replaced with fresh medium supplemented with the drug every 3 days. Mean *FXN* mRNA levels of each sample were normalised to *HPRT* mRNA levels*.* Values were expressed as a ratio to the vehicle treated samples of normal fibroblasts at the corresponding time point. **(E)** Dipstick immunoassay of frataxin protein in primary fibroblasts (FRDA, YG8sR and normal, Y47R) following synergistic treatment with BIX01294 (100nM) and GSK126 (2µM) for different time points. Values are expressed as percentage of the vehicle treated samples of normal fibroblasts at the corresponding time point. Error bars indicate SEM and values represent mean ±SEM (*n*=3). Asterisks indicate significant differences between drug and vehicle treated cell lines, assessed by unpaired two-tailed Student’s *t*-test (**P*<0.05, ***P*<0.01, ****P*<0.001).

**Supplementary Figure 2** qRT-PCR analysis indicating the relative *FXN* mRNA levels following treatment with BIX01294 (100nM) and GSK126 (2µM) individually and synergistically in human primary fibroblasts (FRDA, GM04078 and normal, GM07492). Each result displayed is the mean of two independent experiments and the *FXN* mRNA levels of each sample were normalised to *HPRT* mRNA levels*.* The values were expressed as a ratio to the vehicle treated samples of normal fibroblasts. Error bars indicate SEM and values represent mean ±SEM (*n*=3). Asterisks indicate significant differences between drug and vehicle treated cell lines, assessed by unpaired two-tailed Student’s *t*-test (**P*<0.05, ****P*<0.001).
